# Landscape-Scale Disturbances Modified Bird Community Dynamics in Successional Forest Environment

**DOI:** 10.1371/journal.pone.0081358

**Published:** 2013-11-25

**Authors:** Qing Zhao, Ermias T. Azeria, Mélanie-Louise Le Blanc, Jérôme Lemaître, Daniel Fortin

**Affiliations:** 1 Département de Biologie, Université Laval, Québec, Québec, Canada; 2 Department of Biological Sciences, University of Alberta, Edmonton, Alberta, Canada; Liverpool John Moores University, United Kingdom

## Abstract

Ecosystem-based forest management strives to develop silvicultural practices that best emulate natural disturbances such as wildfire to conserve biodiversity representative of natural forest ecosystems. Yet, current logging practices alter forest structure and reduce the proportion of old-growth forest and, consequently, can exert long-term effects on the dynamics of forest biota. The stand- and landscape-scale factors driving bird community dynamics in post-disturbance environment remain poorly understood. In this study, we examined bird community dynamics along successional gradients in boreal ecosystems originating from fire and logging in landscapes dominated by old-growth forest. We tested if bird species richness and community compositions in clear-cutting stands became comparable to those in natural stands after 70 years, and identified the relative contributions of stand- and landscape-scale forest attributes in bird community dynamics. Based on records of bird occurrences at 185 field sites in natural and clearcutting stands, we demonstrate that (1) both forest structures and bird communities underwent evident changes along successional gradients in post-clearcutting environment; (2) bird species richness and community composition in 60- to 70-years-old clearcutting stands still differed from those in 50- to 79-years-old natural stands, in spite of the fact that most forest attributes of clearcutting stands became comparable to those of natural stands after 40 years; and (3) landscape disturbances contributed more than stand characteristics in explaining the lack of convergence of mature forest species, residents, and short-distance migrants in post-clearcutting environment. Our study points out that more regards should be paid to improve the landscape configuration of the managed forests, and implies that old-growth forest retention within logged areas, combined with selection cutting and prolonged logging rotations, can better emulate fire and alleviate forest harvesting effects on bird community assemblages typical of natural boreal ecosystem.

## Introduction

Sustainable forest management strategies strive to maintain the biodiversity of forest ecosystems [1], [2], by such means as developing forestry practices that best emulate natural disturbance regimes [3], [4]. In boreal forests, wildfire is a key disturbance agent that generates and maintains habitat heterogeneity and complexity and, thus, biodiversity [5]. For this reason, it is thought that forest management strategies that emulate forest fire, such as logging with rotation periods with lengths similar to fire cycles, should be able to maintain the biodiversity in the region [6]. Yet, there are still fundamental differences between logging and forest fire disturbance. Logging, particularly the widely practiced technique of clear-cutting, alters stand structures by removing most of the standing trees and snags, which are key structural elements in post-fire conditions [7]. Further, fire is unpredictable in terms of its timing, location and intensity, thereby resulting in a forest landscape dominated by old-growth stands [8]. In contrast, the disturbance of logging is more regular and its application greatly reduces the proportion of old-growth stands [8], [9].

Clearly, such differences between logging- and fire-originated forests will have significant effects on the assemblage and dynamics of the forest fauna. Indeed, birds are known to be sensitive to the variation in habitat characteristics and successional stages [10] and several studies have indicated substantial differences in bird community composition between logging- and fire-originated forests, particularly in the early stages following disturbances [11], [12]. These studies also point out that the disturbance of logging is more likely to affect residents and species typically associated with mature forest than migrants and generalists [8], [13], [14]. Such group-specific response to disturbances may be obscured by highly summarized biodiversity attributes such as total richness, and failing to distinguish group-specific patterns could mislead assessments of disturbance effects and the decisions made for habitat management [15], [16].

An additional and more fundamental question for long-term conservation of biodiversity is whether an altered community in managed forests would approach the status of a natural community after a period of time. Disturbances, whether they are natural or anthropogenic, typically lead to the abrupt loss of biomass or biodiversity, but they also can generate novel environments that trigger ecological succession [17]. The characterisation of community dynamics in successional systems can aid in assessing the long-term effects of disturbance on biodiversity patterns and, thus, holds significant meaning in conservation biology [18]. However, the long-term effects of logging on bird communities have not been fully understood [8]. In particular, few studies have tried to understand the factors driving bird community dynamics in post-disturbance environment at both stand and landscape scale [13].

The current study characterises bird community dynamics in boreal forests originating from fire and logging in the Côte-Nord region of Québec, Canada. This region has a relatively long fire cycle, which ranges from 270 years in the west to >500 years in the east [19]. In the long absence of fire, the forest landscape is dominated by old-growth forest where small-scale gap dynamics that are driven by windthrow, insects, and forest pathogens have created an irregular forest structure [20]. Although clear-cutting is the most common silvicultural practice in this region [21], this silvicultural practice undoubtedly would remove standing trees and snags, reduce the proportion of old-growth stands, and regenerate to an even-aged forest stands rather than promote development of an irregular forest landscape mosaic [9]. However, the long-term effects of current forest management strategies on the bird communities are not fully understood. Our study aims to (1) examine the post-disturbance dynamics of bird communities along successional gradients in natural and clearcutting forests; (2) test if bird community attributes in an area that has been subjected to clear-cutting became comparable to those in natural forests after 70 years; and (3) identify the relative contributions of stand- and landscape-scale forest attributes in bird community dynamics.

## Materials and Methods

### Study Area

The study was conducted in boreal forest of the Côte-Nord region of Québec, Canada (49°–52°N, 65°–70°W). The region is characterized by a humid climate, where mean annual temperature ranges from −2.5 to 0.0°C and annual precipitation ranges from 1000 to 1400 mm, with 35% falling as snow [22]. Because of the abundant precipitation, the area is characterized by a long fire cycle (average length >270 years), which in turn leads to landscapes dominated by old-growth stands [19]. Dominant tree species include black spruce (*Picea mariana* (Mill.) BSP) and balsam fir (*Abies balsamea* (L.) Mill.). Several other tree species are frequently observed, including white spruce (*Picea glauca* (Moench) Voss), white or paper birch (*Betula papyrifera* Marsh.), trembling aspen (*Populus tremuloides* Michx.), and jack pine (*Pinus banksiana* Lamb.) [22]. More details concerning the characteristics of the study area could be found in previous studies [19], [23].

### Sampling Design and Data Collection

**Figure 1:**
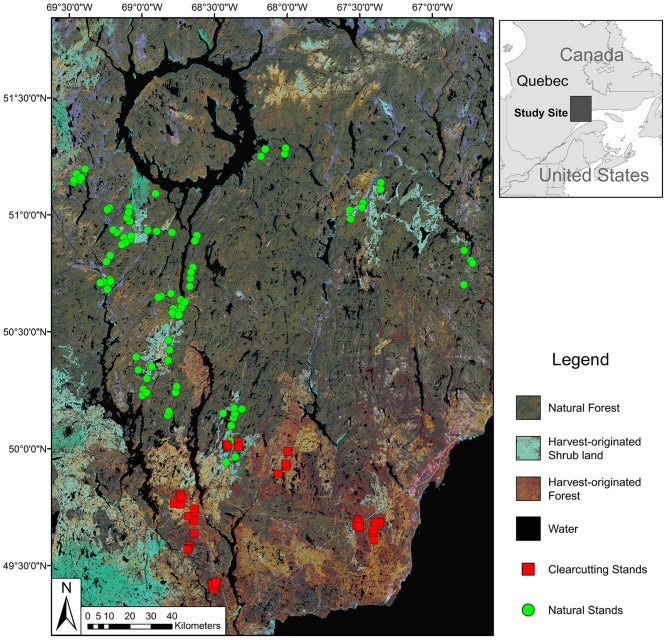
Study area and the locations of sampling sites. The sampling sites are distributed in a boreal forest of the Côte-Nord region of Québec, Canada.

Our sampling sites were distributed in natural forest stands versus stands originated from clearcutting (hereafter clearcutting stands). The natural stands consist of forest stands that originated from fire and which had not been logged. The ages of the natural stands ranged from 50 to 225 years (mean  = 135 years), of which 65% were >120-years-old [16], [24]. To determine the ages of natural stands, we conducted increment-borer sampling [25] on five trees (two dominant trees, one co-dominant tree, one intermediate tree, and one oppressed tree) for each stand. Samples were collected at 1m height above ground, and were analysed later in laboratory. Only the ages of the dominant trees were used to determine the ages of the stands. The natural stands were further classified into three age classes (i.e. 50–79, 80–120, and >120 years; hereafter N-I, N-III, and N-III, respectively). The clearcutting stands were cut with advance growth protection and <10% of tree retention, with stand age ranging from 5- to 70-years-old following harvest [26]. There were more than 52,000 clearcutting stands (area: 11.7±10.4 ha) during the survey period. The ages of these clearcutting stands were obtained from a forest inventory map, which contained the information of the time for each forest harvesting activity. The clearcutting stands were further classified into four age classes (i.e. 5–19, 20–39, 40–59, and 60–70 years; hereafter C-1, C-2, C-3, and C-4, respectively). The sampling was conducted at 88 sites in the natural stands and 97 sites in the clearcutting stands (Fig. 1). More details concerning the number of sampling sites for each age class could be found in Table 1.

**Table 1:** Number of sampling sites for each age class of natural and clearcutting stands.

Stand origin	Age class (yr)	Abbreviation	Number of sampling sites
Natural	50–79	N-I	9
	80–120	N-II	21
	>120	N-III	58
Clearcutting	5–19	C-1	23
	20–39	C-2	23
	40–59	C-3	24
	60–70	C-4	27

We surveyed breeding birds using the fixed-radius point count method [27] during the breeding seasons (June-July) from 2004 to 2007. Each sampling site was >100 m from the edge of the forest and >150 m from any other site. All sites were visited three times, i.e., during the early, middle, and late period of the breeding season, to maximize the probability of detection. Each sampling site was visited in only one of the years. Surveys were carried during the morning between 0500 and 1000 h, in the absence of wind or heavy rain. On each visit, all birds that were observed or heard within 50 m were recorded for 10 minutes.

We characterized sampling sites at the stand and landscape scales. At the stand scale, we established a 400 m^2^ circular plot (radius: 11.3 m) centred on each sampling site and recorded the diameter at breast height (DBH) of every living tree (species identified) and snag having DBH ≥9 cm. Then we calculated the basal areas of conifers and snags based on these measures. We also treated living trees with DBH <9 cm as saplings and counted the number of them within each plot. Then we calculated the sapling densities. We also measured canopy covers using spherical densiometer. These variables were chosen to represent stand-level forest characteristics because previous studies conducted in the same area have shown that these variables can explain the majority of forest structure [28] and are related to understory plant composition [23].

At the landscape scale, we used forest inventory maps, managed with ArcGIS 10 (Environmental Systems Research Inst., Redlands, CA, USA), to characterize the landscape mosaic at different spatial scales. In the forest inventory maps stands were digitalized as polygons which constituted baseline information such as stand origin (natural vs. forest harvest) and stand age for the planning of logging operations by forestry companies. We then quantified the proportion of logged areas within buffer areas centred on the sampling sites at four spatial scales, each being matched to a radius of 200 m, 500 m, 1 km, and 2 km, respectively.

### Data Analysis

We compiled bird species presence-absence in each sampling site. First, we examined whether stand structure (characterized by four stand-level variables including basal area of conifer, canopy cover, sapling density, and basal area of snag) differed among any age classes of natural and clearcutting stands, using one-way Multivariate Analysis of Variance (MANOVA). Age class included three categories of natural stand and four categories of clearcutting stand (see above). If a significant difference in stand structure was detected, we conducted one-way Analysis of Variance (ANOVA) for different stand characteristics to examine which ones differed among age classes. Holm–Bonferroni method was applied in the ANOVAs to reduce the probability of making type I error so that the family-wise type I error probability was less than 0.05. Each ANOVA was followed by Tukey multiple comparisons to examine (1) if there was a difference between individual age classes of clearcutting stands, and (2) if there was a difference between 60- to 70-years-old clearcutting (C-4) stands and 50- to 79-years-old natural (N-I) stands. A difference between any age classes of clearcutting stands would indicate evident changes in stand structure along successional gradients in post-clearcutting environment, and a difference between C-4 and N-I stands would indicate poor comparability in stand structure between clearcutting and natural stands of similar ages.

We further conducted constrained ordination to understand the relative positions of sampling sites in a multi-dimensional space characterized by environmental variables, including stand characteristics, landscape disturbances (i.e. the proportion of disturbed area at 200 m, 500 m, 1 km, and 2 km), and geographic positions (i.e. latitude and longitude). Community variations across sampling sites were projected to a multi-dimensional space “constrained” by these environmental variables. Each of the ordination axes was a linear combination of the environmental variables and all the ordination axes were orthogonal to each other.

Second, we compared mean species richness (alpha diversity) between different age classes of natural and clearcutting stands. To do so, we first applied a hierarchical modelling approach to estimate species richness for each site while accounting for imperfect detection of individual species [29]. In this approach we assumed different detection probability for each species. To account for year- and observer-effect, we assumed different detection probabilities for sites that were visited in different years and surveyed by different observers. Estimated species richness was calculated as the sum of true occupancy probabilities, which would be 1 for species that were detected, and a number between 0 and 1 for species that were not detected. We then conducted one-way ANOVA and Tukey multiple comparisons using the estimated total richness as the dependent variable and age class as the independent variable. Further, we broke down the one-way ANOVA into each subset of species according to their habitat association (i.e., mature forest species, young forest species, shrub land species, and generalists) or migratory status (i.e., residents, short-distance migrants, and Neotropical migrants; Table S1). In each ANOVA the estimated richness of a characteristic group was treated as the dependent variable and age class was the independent variable. Holm–Bonferroni method was applied in the ANOVAs of group richness to ensure that the family-wise type I error probability was less than 0.05. The information of habitat association and migratory status was compiled from existing references [13], [30].

We also examined the compositional differences (beta diversity) among treatments using a non-parametric test, viz., Permutational Multivariate Analysis of Variance (PERMANOVA) [31], based on Sørensen dissimilarity indices. PERMANOVA calculates a Pseudo-*F*-statistic and corresponding *P*-value based on permutations of species compositional data. A significant PERMANOVA Pseudo-*F*-value may indicate a difference in dissimilarity among the treatments, different within-treatment dispersion, or both conditions. We used a principal component ordination plot based on species composition dissimilarity matrix (Sørensen) to visualize compositional differences between treatments and the composition dispersion of treatments from their respective community centroids [28]. In principal component ordinations community variations across sampling sites were projected to a multi-dimensional space. Each of the ordination axes was a linear combination of species presence-absence and all the ordination axes were orthogonal to each other. To test whether or not treatments differed in their within-treatment dispersion (i.e., beta diversity), we performed a complementary analysis, Permutational Analysis of Multivariate Dispersions (PERMDISP) [32]. PERMDISP is a multivariate extension of Levene's test of homogeneity of variance [33]. As is the case of PERMANOVA, PERMDISP generates Pseudo-*F*-statistics based on any distance measure and following permutation of the observations. We permuted the data 1000 times for each test. When significant results were obtained from PERMDISP, we carried out pairwise tests using Tukey multiple comparisons (Tukey's Honestly Significant Difference test).

Third, we conducted multiple regressions and model averaging to examine the relative importance of the environmental variables in explaining total- or group-richness. Because the estimated richness, unlike the observed richness, was not a count number, it was log transformed and Gaussian models were conducted. In each regression analysis, total- or group-richness was treated as the dependent variable, and the environmental variables (see above) were the independent variables. In the model averaging protocol the relative importance of each explanatory variable was calculated as a sum of the Akaike weights over all of the models in which the parameter of interest appears [34]. Variables with model averaging importance great than or equal to 0.95 are considered as significant explanatory variables. We also conducted hierarchical partitioning based on multiple Poisson regression to calculate the independent contribution of environmental variables in explaining the variance of species richness. In this protocol all possible combinations of explanatory variables in a multiple regression setting are jointly considered to identify the most likely causal factors [35]. Hierarchical partitioning employs goodness of fit measures for each of the 2^K^ possible models, in which K is the total number of explanatory variables. The increase in model fit generated by an explanatory variable is estimated by averaging the pairwise improvements of the 2^K−1^ models that include this variable over the 2^K−1^ models without this variable. This process is repeated for K times to partition the variances so that the total independent contribution of each explanatory variable can be estimated. By these means, hierarchical partitioning distinguish those variables that have high independent, not partial, correlation with a response variable from variables that have little independent effect on the response variable. The latter may still have a high pair-wise correlation with the response variable but this is due to joint action with other explanatory variables. We used the log-likelihood of the models as the measures of goodness-of-fit. These two approaches were likely to alleviate multicollinearity problems that were otherwise ignored by using any single-model technique.

All analyses were performed in R2.15.2 (R Development Core Team 2012).

### Ethics Statement

No specific permissions were required for our study area and locations because it was not a protected area or private land under restriction from entering. Our field studies did not involve endangered or protected species. No approval was necessary for the data collection procedure because no captures were done; the study was only observational.

## Results

### Forest Structure in Post-disturbance Environment

**Figure 2:**
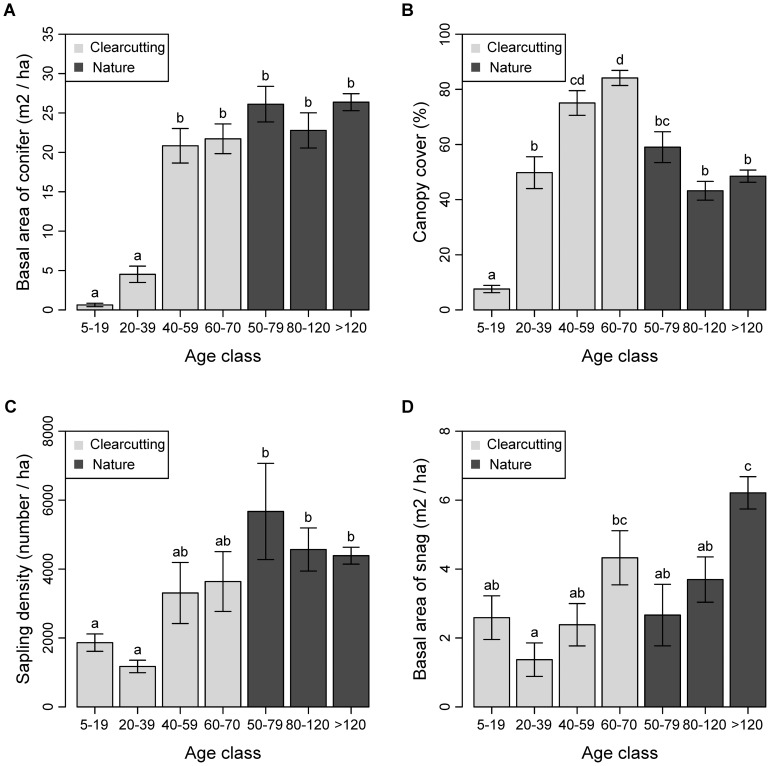
Mean ± SE of stand-scale forest attributes in clearcutting and natural stands. (a) Basal area of conifer, (b) canopy cover, (c) sapling density, and (d) basal area of snag. Age classes that share the same letter (above the bar) are not significantly different in the corresponding stand structure at *α* = 0.05, according to Tukey multiple comparisons.

One-way MANOVA indicated significant difference in stand structure between age classes, regardless of the stand's origin (*F*_24,712_ = 14.96, *P*<0.001). One-way ANOVA, followed by Tukey multiple comparisons, indicated significant increase in basal area of conifer (*F*_6,178_ = 41.34, *P*_Holm–Bonferroni_ <0.001; Fig. 2a) and canopy cover (*F*_6,178_ = 45.97, *P*_Holm–Bonferroni_ <0.001; Fig. 2b) after clearcutting along successional gradients. Tukey multiple comparisons showed that basal area of conifer in 60- to 70-year-old clearcutting stands (hereafter C-4 stands) was indistinguishable to that in 50- to 79-year-old natural stands (hereafter N-I stands; *P* = 0.807), whereas canopy cover was higher in C-4 stands than in N-I stands (*P* = 0.007). In addition, Tukey multiple comparisons showed that C-4 and N-I stands were indistinguishable in their sampling density (*P* = 0.543) and basal area of snag (*P* = 0.846; Fig. 2c, d). Overall, except having higher canopy cover, C-4 stands were comparable with N-I stands in terms of forest structures.

**Figure 3:**
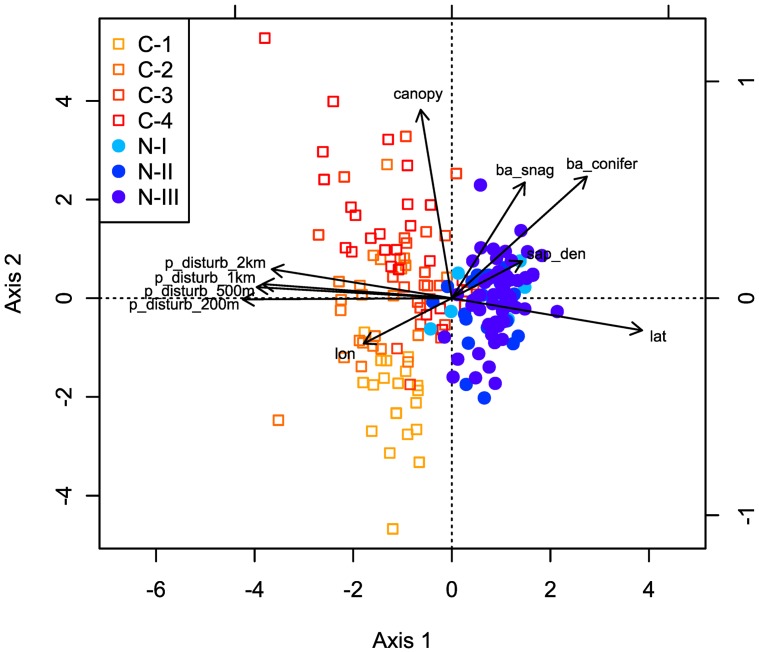
The positions of sampling sites in a constrained ordination space characterised by environmental variables. An arrow's length and its angle represent the strength of the correlation between the corresponding environmental variable and the first two ordination axes. The first axis of the constrained ordination diagram showed latitudinal transition of landscape-level disturbance gradients, and the second axis showed differences in canopy cover across successional gradient in post-clearcutting environment. Environmental variables: lon: longitude, lat: latitude, ba_conifer: basal area of conifer, canopy: canopy cover, sap_den: sapling density, ba_snag: basal area of snag, p_disturb_200 m: proportion of disturbed area at 200 m, p_disturb_500 m: proportion of disturbed area at 500 m, p_disturb_1 km: proportion of disturbed area at 1 km, p_disturb_2 km: proportion of disturbed area at 2 km. Age classes: C-1, C-2, C-3, and C-4: 5–19, 20–39, 40–59, and 60–70 year old clearcutting stands, respectively, N-I, N-II, and N-III: 50–79, 80–120, and >120 year old natural stands, respectively.

The constrained ordination diagram (Fig. 3) showed a clear distinction between the natural stands that had high basal area of conifer, basal area of snag, and sapling density and clearcutting stands that had high proportion of disturbed area from 200 m to 5 km scales. Moreover, the first axis of the constrained ordination diagram showed latitudinal transition of landscape-level disturbance gradients from high latitude where natural stands predominated to low latitude where forest harvesting mainly occurred (also see Fig. 1). The second axis showed differences across successional gradient in post-clearcutting environment, where canopy cover was highest in the 60- to 70-year-old clearcutting stands and lowest in the 5- to 19-year-old clearcutting stands.

### Bird Community Dynamics along Successional Gradients

Seventy bird species were recorded in the study. Among these species 28 (40.0%) were known to be associated with mature forest, 14 (20.0%) with young forest, 12 (17.1%) with shrub land, and 16 (22.9%) were generalists. These species included 13 (18.6%) residents, 23 (32.9%) short-distance migrants, and 34 (48.6%) Neotropical migrants (Table S1).

**Figure 4:**
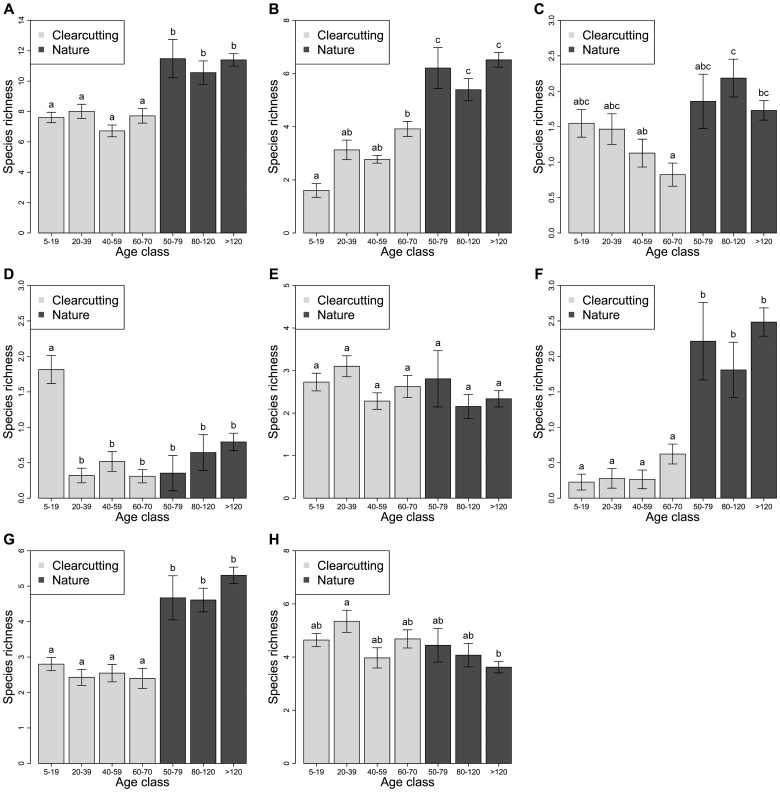
Mean ± SE of species richness in clearcutting and natural stands. (a) All species, (b) mature forest species, (c) young forest species, (d) shrub land species, (e) generalists, (f) residents, (g) short-distance migrants, and (h) Neotropical migrants. Age classes that share the same letter (above the bar) are not significantly different in their species richness at *α* = 0.05, according to Tukey multiple comparisons.

We compared species richness between different age classes of clearcutting and natural stands. One-way ANOVA indicated that total richness differed between age classes (*F*_6,178_ = 14.30, *P*<0.001), and Tukey multiple comparison indicated that total richness did not change after clearcutting (*P*>0.544), and remained lower than that in natural stands after 60 to 70 years (*P*<0.001; Fig. 4a). Further, we found that different characteristic groups differ in their post-logging dynamics along successional gradients. The richness of species associated with mature forest increased after clearcutting along successional gradients (*F*_6,178_ = 33.86, *P*_Holm–Bonferroni_ <0.001; Tukey multiple comparison: *P*<0.001; Fig. 4b); however, it was lower in C-4 stands than in N-I stands (Tukey multiple comparison: *P*<0.001). The richness of species associated with shrub land was high immediately after clearcutting, and became as low as that in natural stands after 20 years (*F*_6,178_ = 9.14, *P*_Holm–Bonferroni_ <0.001; Tukey multiple comparison: *P* = 1.000; Fig. 4d). The richness of residents and short-distance migrants in clearcutting stands remained lower than that in natural stands after 60 to 70 years (residents: *F*_6,178_ = 21.29, *P*_Holm–Bonferroni_ <0.001; Tukey multiple comparison: *P*<0.001; Fig. 4f; short-distance migrants: *F*_6,178_ = 24.77, *P*_Holm–Bonferroni_ <0.001; Tukey multiple comparison: *P*<0.001; Fig. 4g), as they did not change after clearcutting (residents: Tukey multiple comparison: *P*>0.906; short-distance migrants: Tukey multiple comparison: *P*>0.979). There were differences in species richness between age classes for young forest species (*F*_6,178_ = 85.23, *P*_Holm–Bonferroni_ <0.001), and the richness of young forest species was lower in 40- to 59-year-old or 60- to 70-year-old clearcutting stands than in 80- to 120-year-old natural stands (Tukey multiple comparison: *P*<0.001), and lower in 60- to 70-year-old clearcutting stands than in natural stands that were older than 120 years (Tukey multiple comparison: *P*<0.001; Fig. 4c). There were differences in species richness between age classes for Neotropical migrants (*F*_6,178_ = 55.33, *P*_Holm–Bonferroni_ <0.001), and the richness of Neotropical migrants was higher in 20- to 39-year-old clearcutting stands than in natural stands that were older than 120 years (Tukey multiple comparison: *P*<0.001; Fig. 4h). Tukey multiple comparison did not show significant difference in species richness between C-4 and N-I stands for young forest species (*P* = 0.131) and Neotropical migrants (*P* = 0.999). There was no difference in the richness of generalists between any age classes (*F*_6,178_ = 1.84, *P*_Holm–Bonferroni_  = 0.906; Fig. 4e).

**Figure 5:**
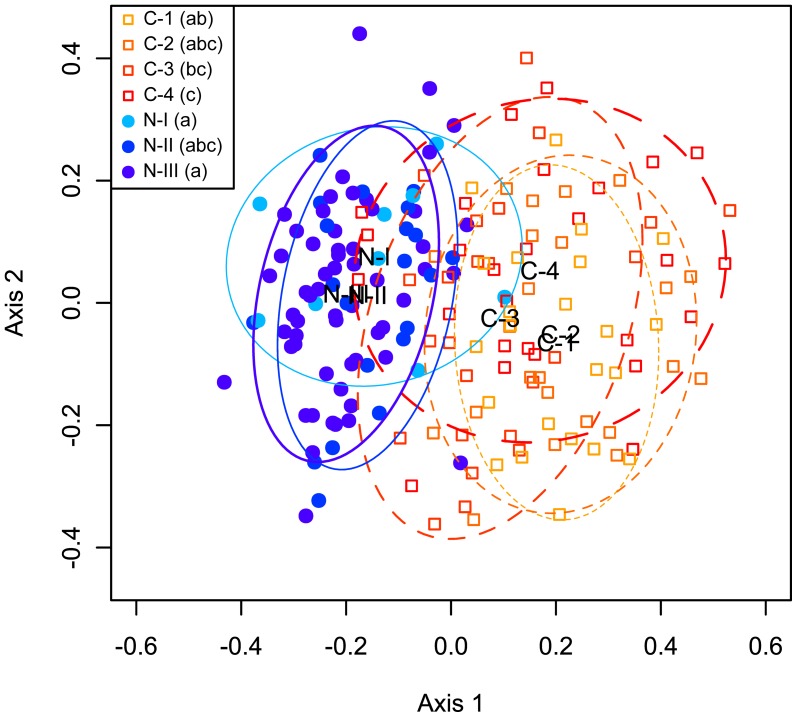
Principal component ordinations represent dissimilarity in assemblage patterns. The principal component ordinations are based on Sørensen indices. The dissimilarity in assemblage patterns between different age classes of clearcutting stands versus natural stands is shown. The first principal ordination axis was along the disturbance gradient and explained 38.7% of assemblage variation, and the second principal ordination axis as along the successional gradient and explained 14.3% of assemblage variation. The ellipses enclose 80% of the variability in compositional differences accounted by the first two ordination axes. Age classes that share the same letter (in the brackets) are not significantly different in their dispersion at *α* = 0.05, according to Tukey multiple comparisons. Age classes: C-1, C-2, C-3, and C-4: 5–19, 20–39, 40–59, and 60–70 year old clearcutting stands, respectively, N-I, N-II, and N-III: 50–79, 80–120, and >120 year old natural stands, respectively.

Bird composition significantly differed between different age classes of clearcutting and natural stands (PERMANOVA: Pseudo-*F*_6,178_ = 9.79, *P*<0.001; Fig. 5). Such difference might indicate difference in composition between age-classes, different within-age-class dispersion, or both conditions. Principal component ordination showed that the bird communities were structured across disturbance gradients (Fig. 5), with the bird communities in natural stands being clustered tightly and separated from the bird communities in clear cutting stands along the first principal ordination axis (which explained 38.7% of assemblage variation). The second principal ordination axis (which explained 14.3% of assemblage variation) was along successional gradient which indicated slight difference among age classes of clearcutting stands. Clearcutting stands and natural stands differed in bird composition because their centroids were mostly outside each other's 80% confidence interval. This result indicated that some species common in natural stands were rare in clearcutting stands, and verse versa (more details see Table S1).

Furthermore, we found significant differences in the dispersions among different age classes of clearcutting and natural stands (PERMDISP: Pseudo- *F*_6,178_ = 6.36, *P*<0.001), indicating that some age classes had higher variability in bird composition than others. Tukey multiple comparisons showed that C-4 clearcutting stands had higher dispersion than 5- to 19-year-old (C-1) clearcutting stands (C-1: 0.37, C-4: 0.46), whereas the centroid of bird composition did not show a significant shift in post-clearcutting environment (Fig. 5), indicating that bird composition did not change after clearcutting, although bird composition was more variable between sites in older clearcutting stands than in younger clearcutting stands. Further, the bird composition in C-4 stands was significantly different from that in N-I stands in terms of both composition centroid (Fig. 5) and dispersion (N-I: 0.34) indicating that C-4 stands harboured a different set of species than N-I stands, and the species composition in C-4 stands was more variable than that in N-I stands. The largest differences of species incidence between N-I and C-4 stands existed for species including Yellow-rumped Warbler (66.7%), Swainson's Thrush (40.7%), Grey Jay (40.7%), Boreal Chickadee (40.7%), and Pine Siskin (40.7%), which were observed more frequently in natural stands.

### Relative Contribution of Stand- and Landscape-scale Attributes

Multiple regression and model averaging showed that the proportion of disturbed areas at 200 m scale was the only significant variable in explaining total-richness, and total-richness tended to be higher in less disturbed areas (Table S2). Further, hierarchical partitioning revealed that the relative independent contribution of landscape disturbances (i.e. the proportion of logged areas within 200 m, 500 m, 1 km, and 2 km) in explaining the variance of total-richness was 72.2%, which was higher than the 13.8% relative independent contribution of local stand characteristics (i.e. basal area of conifer, canopy cover, sapling density, and basal area of snag). It held true that landscape disturbances contributed more than stand characteristics in explaining the variance of total-richness when limiting the analysis to C-4 and N-I stands (landscape disturbance: 57.2%, stand characteristic: 24.6%; Table S2).

Landscape disturbances contributed more than stand characteristics in explaining the richness of mature forest species, residents, and short-distance migrants. The proportion of disturbed areas at 200 m scale was also significantly negatively correlated with the richness of mature forest species, residents, and short-distance migrants. In addition, canopy cover was significantly negatively correlated with the richness of short-distance migrants. Landscape disturbances had 61.5% to 73.1% relative independent contribution in explaining the richness of mature forest species, residents, and short-distance migrants, which were higher than the 11.3% to 22.3% relative independent contribution of local stand characteristics; this held true when limiting the analyses to C-4 and N-I stands.

In contrast, stand characteristics contributed more than landscape disturbances in explaining the richness of young forest species, shrub land species, generalist, and Neotropical migrants. Canopy cover was significantly negatively correlated with the richness of shrub land species, and stand characteristics had greater independent contribution than landscape disturbance in explaining the variance of the richness of this group of species (stand characteristic: 77.8%, landscape disturbance: 12.5%). Likewise, basal area of conifer was significantly negatively correlated with the richness of generalists and Neotropical migrants, and stand characteristics had greater independent contribution than landscape disturbance in explaining the variance of these two groups of species (stand characteristic: 37.2% to 49.0%, landscape disturbance: 27.2% to 31.2%). Canopy cover, basal area of snag, and the proportion of disturbed area at 200 m scale were significantly negatively correlated with the richness of young forest species, and stand characteristics had greater independent contribution than landscape disturbance in explaining the variance of the richness of young forest species, although the difference seemed to be less evident (stand characteristic: 44.6%, landscape disturbance: 42.2%).

Note that latitude and longitude were included in these analyses to control for the effects of geographic positions. Thus the relative independent contributions of stand characteristics and landscape disturbance reported above were not a result of the geographic positions of the sampling sites. More details concerning the log-likelihood and Akaike information criterion (AIC) values of the models could be seen in Table S3.

## Discussion

Our study demonstrates that both forest structures and bird communities underwent evident changes along successional gradients in post-clearcutting environment. However, bird species richness and community composition in 60- to 70-years-old clearcutting stands still differed from those in 50- to 79-years-old natural stands, in spite of the fact that most forest attributes of clearcutting stands became comparable to those of natural stands after 40 years. Although both stand characteristics and landscape disturbances contribute in explaining the variance of species richness, landscape disturbances contribute much more than stand characteristics in explaining the variance of the richness of mature forest species, residents, and short-distance migrants, which are the species groups whose richness were maintained at lower levels in clearcutting stands than in natural stands after 60 to 70 years.

### Post-disturbance Dynamics of Bird Communities and Group-specific Patterns

Our study shows that the early post-clearcutting stands supported bird assemblages with low variability in species composition, which may reflect the fact that clearcutting creates a relatively homogeneous habitat for the few bird species that are typically associated with disturbed, early-successional environments [12]. As succession progresses, however, the sites may become more heterogeneous and can support both early- and late-successional species, which may explain the higher variability in species composition in the 60- to 70-year-old clearcutting stands than in the early post-clearcutting stands. The natural stands above 50 years old, on the other hand, showed different levels of variability in bird community composition, and such difference does not seem to be related to stand age. This may be because that the habitat heterogeneity of old-growth forests is mainly driven by small-scale disturbances such as windthrow, insects, and forest pathogens, and the occurrences of these factors are, unlike that of forest fire, unrelated to stand age [20].

Although bird community composition underwent evident changes along successional gradients in post-clearcutting environment, such changes mainly reflected the increase in compositional variability rather than a shift of compositional centroid. In addition, the centroid of bird community composition in clearcutting stands remained different than that in natural stands after 60 to 70 years. Such finding is consistent with the group-specific patterns in species richness that, although the richness of young forest species, shrub land species, generalists, and Neotropical migrants in late post-clearcutting stands were comparable to those in natural stands, the richness of mature forest species, residents, and short-distance migrants in clearcutting stands remained lower than those in natural stands after 60 to 70 years.

It is increasingly recognized in the ecological literature that different groups of species may respond differently, and even in opposite manners, to the same underlying causal factor [36], and this general idea has been supported by studies focusing on bird community in response to forest harvesting [8], [13]. These studies show that mature forest species and residents tend to decline in areas under anthropogenic disturbances, and these declining species share certain life history traits such as large home range, dependence on decaying wood for foraging, and cavity nesting. [14]. Our study shows that mature forest species, residents, and short-distance migrants were negatively affected by clearcutting, whereas shrub land species tended to thrive in early successional forest after clearcutting, which is consistent with previous studies [8], [13]. In particular, mature forest species and shrub land species showed opposite trends in their post-disturbance dynamics, which would be obscured if total richness was the only biodiversity attribute that was examined.

Some of the previous studies are apt to classify species into groups according to their migratory status [8]. Although such classification is proved to be adequate in revealing group-specific response to disturbances, our study shows that a classification of species according to their habitat associations may be more informative in revealing group-specific dynamics along successional gradients [13]. Overall, our study demonstrates that caution should be exercised when choosing biodiversity attributes to examine biodiversity patterns.

### Scale-dependent Long-term Effects of Clearcutting on Bird Communities

It is evident that logged areas support bird communities that are easily distinguishable from those of natural forest [11], [12], [26], but it is also important to determine, in the context of ecosystem-based management, how long it takes for species assemblages to regain their pre-logging characteristics. However, this has been seldom demonstrated [8], [13].

Our study shows that neither species richness nor community composition of bird in the clearcutting stands approached that of natural stands, even though 70 years had elapsed following harvest. Further, our study shows that such differences mainly reflected the lack of convergence of some, but not all, characteristic groups, namely mature forest species, residents, and short-distance migrants. Other studies likewise found that abundances of bird species associated with early-successional habitats were maintained at high levels, whereas abundances of mature forest birds remained low in managed forest landscapes [13]. However, our study shows that most forest attributes of clearcutting stands became comparable to those of natural stands after 40 years. The only forest attribute that was different between 60- to 70-year-old clearcutting stands and natural stands was canopy cover, which was low in early post-clearcutting stands, but increased along successional gradients and became higher than that in natural stands after 60 to 70 years. Although the dense canopy cover in late post-clearcutting stands seems to be consistent with the finding of previous studies that clearcutting tends to regenerate to even-aged forest stands, we find it is hard to link this and other stand characteristics to the phenomenon that the variability of bird community composition was higher, and the species richness of mature forest species and residents was lower in late post-clearcutting stands than in natural stands.

As a matter of fact, stand characteristics could only explain a small proportion of variance of species richness in compare with landscape disturbances. This is particularly true for mature forest species, residents, and short-distance migrants, which are the groups that lack of convergence in post-clearcutting environment. Thus our study shows that the effects of clearcutting are at landscape scale. In particular, the proportion of disturbed area at 200 m to 1 km scales negatively affected the richness of residents, indicating that the effects of logging may well extend to 1 km. Further, the result holds when the effect of stand age is controlled by limiting the analysis to 60- to 70-year-old clearcutting stands and 50- to 79-year-old natural stands. Although late post-clearcutting stands largely regain their pre-logging characteristics in term of forest structure, they are embedded in a disturbed matrix and probably suffer from matrix-effects from surrounding disturbed areas [14], [37]. Thus our results indicate that the effects of logging may last much longer than the time needed for post-clearcutting stands to regain their pre-logging forest structure because of the continued disturbances at landscape scale.

### Conservation Implications

Our study shows that, in landscapes dominated by old-growth forest, clearcutting would have a profound influence on bird community composition and species richness, and ultimately, on ecosystem functioning [38]. The current forestry practices would therefore violate some fundamental principles of ecosystem-based management.

Because forest stands experienced clearcutting are highly distinguishable from natural stands in terms of forest structure and biodiversity patterns, silvicultural practices have attempted replacing clearcutting with harvesting system such as selection cutting that may better protect stand structure [26]. However, even though selection cutting can better simulate forest fire in its early post-disturbance status, such prescription is not necessarily sufficient in reducing the proportion of disturbed area at landscape, which is suggested to be important by our study. Because the history of selection cutting is relatively short, the long-term effects of selection cutting at landscape scale need to be investigated in the future.

Another stream of prescription to improve the current forest practices is the one that suggests prolonging logging rotation. Socioeconomic values create strong incentives for reducing as much possible the time required before a given forest stand can be reharvested. A rotation cycle as short as 40 years has been commonly suggested [39]. Previous studies suggest that the long-term effects of logging may persist in the managed forests because of relative shorter length of logging rotations compared with fire cycles [40]. Our study shows that the effects of logging could persist in the managed forests for at least 70 years. However, our study also shows that 40 years may be sufficient for post-logging stands to regain their pre-logging forest structure. Thus the long-term effects of logging in the managed forests are not because logging rotation is short but because logging is more regular than natural disturbances such as fire in terms of timing, location, and intensity. Because of the regular characteristic of logging, the loss of undisturbed old-growth forest at landscape scale has not stopped in the last century and seems to be permanent [8]. This is particularly severe in areas with long fire cycles and, thus, high proportion of old-growth forests [19]. Prolonging logging rotation may not be a sufficient solution to this problem if the regular characteristic of logging remains unchanged.

Studies suggest that old-growth forest stands should be retained within the logged area [8]. Also, these studies have shown that patches of old-growth forest are necessary for conserving resident species that are restricted to mature forest. Consequently, the size of the patches should not be smaller than their required home ranges, which are often relatively large [11]. Our study supports such suggestion but provides another ration for it: retaining large patches of old-growth forest can reduce the proportion of disturbed area at landscape scale. Further, because our study suggests that the effects of logging may well extend to 1 km, the size of old-growth forest should be larger than this scale so that it can buffer the matrix effect from surrounding disturbed areas.

Our study provides information that can aid the development of forest management strategies that should be effective in alleviating effects of forest harvesting on bird assemblages in boreal landscapes dominated by old-growth forests. The basic principle is to develop forest practices that can well simulate natural disturbance regimes at both stand and landscape scales. So far many prescriptions have been put forward and each of them can help to achieve the above goal in one way or another. However, it is highly needed to integrate these prescriptions so that their positive effects could be combined. In particular, more regards should be paid to improve the landscape configuration of the managed areas.

## Supporting Information

Table S1 The proportion (in %) of sampling sites in which each bird species was recorded in natural and clearcutting stands, and the species' habitat association and migratory status.
(DOCX)

Table S2 The relative importance and independent contribution of environmental variables in explaining the variance of total- and group-richness calculated from (a) all data and (b) 60- to 70-year-old clearcutting stands and 50- to 79-year-old natural stands only.
(DOCX)

Table S3 The basic information of models used in model averaging and hierarchical partitioning procedures for (a) all data and (b) 60- to 70-year-old clearcutting stands and 50- to 79-year-old natural stands only.
(DOCX)
