# Supplementary material for: Landscape-Scale Disturbances Modified Bird Community Dynamics in Successional Forest Environment
Source: PLoS One. 2013 Nov 25;8(11):e81358. doi: 10.1371/journal.pone.0081358 (PMC3839899; doi:10.1371/journal.pone.0081358)
Supplement: Table S1 — The proportion (in %) of sampling sites in which each bird species was recorded in natural and clearcutting stands, and the species' habitat association and migratory status. (DOCX) [file pone.0081358.s001.docx]

| Common English name | Latin name | Habitat association^a^ | Migratory status^b^ | Study area | Natural stand^c^ | | | | Clear-cutting stand^c^ | | | | |
| --- | --- | --- | --- | --- | --- | --- | --- | --- | --- | --- | --- | --- | --- |
|  |  |  |  |  | Total | N-I | N-II | N-III | Total | C-1 | C-2 | C-3 | C-4 |
| White-throated Sparrow | *Zonotrichia albicollis* | GEN | SDM | 80.0 | 84.1 | 77.8 | 81.0 | 86.2 | 76.3 | 100.0 | 100.0 | 62.5 | 48.1 |
| Ruby-crowned Kinglet | *Regulus calendula* | MAF | NEO | 79.5 | 92.1 | 100.0 | 81.0 | 94.8 | 68.0 | 56.5 | 69.6 | 79.2 | 66.7 |
| Swainson's Thrush | *Catharus ustulatus* | MAF | NEO | 61.1 | 83.0 | 88.9 | 76.2 | 84.5 | 41.2 | 21.7 | 39.1 | 54.2 | 48.1 |
| Dark-eyed Junco | *Junco hyemalis* | YOF | SDM | 56.2 | 77.3 | 55.6 | 76.2 | 81.0 | 37.1 | 30.4 | 39.1 | 50.0 | 29.6 |
| Tennessee Warbler | *Vermivora peregrina* | GEN | NEO | 44.9 | 21.6 | 55.6 | 23.8 | 15.5 | 66.0 | 47.8 | 69.6 | 75.0 | 70.4 |
| Winter Wren | *Troglodytes troglodytes* | MAF | SDM | 43.2 | 62.5 | 66.7 | 57.1 | 63.8 | 25.8 | 26.1 | 26.1 | 12.5 | 37.0 |
| Yellow-rumped Warbler | *Dendroica coronata* | MAF | SDM | 38.4 | 71.6 | 77.8 | 61.9 | 74.1 | 8.3 | 0.0 | 4.3 | 16.7 | 11.1 |
| Magnolia Warbler | *Dendroica magnolia* | GEN | NEO | 34.6 | 27.3 | 44.4 | 23.8 | 25.9 | 41.2 | 47.8 | 43.5 | 25.0 | 48.1 |
| Hermit Thrush | *Catharus guttatus* | YOF | SDM | 29.7 | 29.6 | 22.2 | 33.3 | 29.3 | 29.9 | 60.9 | 21.7 | 16.7 | 22.2 |
| Red-breasted Nuthatch | *Sitta canadensis* | MAF | RES | 28.7 | 45.5 | 44.4 | 42.9 | 46.6 | 13.4 | 4.3 | 13.0 | 4.2 | 29.6 |
| Nashville Warbler | *Vermivora ruficapilla* | YOF | NEO | 28.1 | 28.4 | 33.3 | 42.9 | 22.4 | 27.8 | 43.5 | 26.1 | 29.2 | 14.8 |
| Grey Jay | *Perisoreus canadensis* | MAF | RES | 25.4 | 47.7 | 44.4 | 33.3 | 53.4 | 5.2 | 4.3 | 4.3 | 8.3 | 3.7 |
| Golden-crowned Kinglet | *Regulus satrapa* | MAF | SDM | 24.9 | 33.0 | 55.6 | 23.8 | 32.8 | 17.5 | 4.3 | 4.3 | 29.2 | 29.6 |
| American Robin | *Turdus migratorius* | SHR | SDM | 22.2 | 26.1 | 22.2 | 19.0 | 29.3 | 18.6 | 8.7 | 13.0 | 25.0 | 25.9 |
| Boreal Chickadee | *Poecile hudsonicus* | MAF | RES | 21.1 | 40.9 | 44.4 | 38.1 | 41.4 | 3.1 | 0.0 | 4.3 | 4.2 | 3.7 |
| American Redstart | *Setophaga ruticilla* | GEN | NEO | 20.0 | 3.4 | 0.0 | 4.8 | 3.4 | 35.1 | 39.1 | 43.5 | 16.7 | 40.7 |
| Alder Flycatcher | *Empidonax alnorum* | SHR | NEO | 18.9 | 11.4 | 11.1 | 9.5 | 12.1 | 25.8 | 87.0 | 13.0 | 4.2 | 3.7 |
| Pine Siskin | *Carduelis pinus* | GEN | SDM | 16.8 | 31.8 | 44.4 | 33.3 | 29.3 | 3.1 | 0.0 | 0.0 | 8.3 | 3.7 |
| Black-backed Woodpecker | *Picoides arcticus* | MAF | RES | 15.1 | 30.7 | 33.3 | 19.0 | 34.5 | 1.0 | 0.0 | 0.0 | 0.0 | 3.7 |
| Red-eyed Vireo | *Vireo olivaceus* | MAF | NEO | 15.1 | 2.3 | 0.0 | 4.8 | 1.7 | 26.8 | 13.0 | 56.5 | 4.2 | 33.3 |
| Yellow-bellied Flycatcher | *Empidonax flaviventris* | GEN | NEO | 15.1 | 14.8 | 11.1 | 9.5 | 17.2 | 15.5 | 17.4 | 13.0 | 16.7 | 14.8 |
| Bay-breasted Warbler | *Dendroica castanea* | MAF | NEO | 14.1 | 3.4 | 11.1 | 0.0 | 3.4 | 23.7 | 4.3 | 17.4 | 25.0 | 44.4 |
| White-winged Crossbill | *Loxia leucoptera* | MAF | RES | 13.5 | 27.3 | 11.1 | 14.3 | 34.5 | 1.0 | 0.0 | 0.0 | 0.0 | 3.7 |
| Brown Creeper | *Certhia americana* | MAF | SDM | 13.0 | 25.0 | 0.0 | 9.5 | 34.5 | 2.1 | 0.0 | 0.0 | 4.2 | 3.7 |
| Least Flycatcher | *Empidonax minimus* | YOF | NEO | 11.4 | 8.0 | 22.2 | 9.5 | 5.2 | 14.4 | 0.0 | 39.1 | 8.3 | 11.1 |
| Chipping Sparrow | *Spizella passerina* | YOF | NEO | 10.3 | 21.6 | 33.3 | 33.3 | 15.5 | 0.0 | 0.0 | 0.0 | 0.0 | 0.0 |
| American Goldfinch | *Carduelis tristis* | GEN | SDM | 9.7 | 15.9 | 0.0 | 19.0 | 17.2 | 4.1 | 4.3 | 0.0 | 8.3 | 3.7 |
| Lincoln's Sparrow | *Melospiza lincolnii* | SHR | NEO | 8.1 | 6.8 | 0.0 | 9.5 | 6.9 | 9.3 | 34.8 | 0.0 | 4.2 | 0.0 |
| Common Raven | *Corvus corax* | GEN | RES | 7.6 | 15.9 | 33.3 | 4.8 | 17.2 | 0.0 | 0.0 | 0.0 | 0.0 | 0.0 |
| Cape May Warbler | *Dendroica tigrina* | MAF | NEO | 7.0 | 1.1 | 0.0 | 0.0 | 1.7 | 12.4 | 0.0 | 8.7 | 8.3 | 29.6 |
| Northern Waterthrush | *Seiurus noveboracensis* | MAF | NEO | 4.9 | 3.4 | 0.0 | 9.5 | 1.7 | 6.2 | 0.0 | 21.7 | 4.2 | 0.0 |
| Tree Swallow | *Tachycineta bicolor* | SHR | SDM | 4.9 | 4.6 | 0.0 | 4.8 | 5.2 | 5.2 | 21.7 | 0.0 | 0.0 | 0.0 |
| Blackpoll Warbler | *Dendroica striata* | MAF | NEO | 4.3 | 3.4 | 0.0 | 14.3 | 0.0 | 5.2 | 8.7 | 4.3 | 4.2 | 3.7 |
| Black-throated Green Warbler | *Dendroica virens* | MAF | NEO | 4.3 | 6.8 | 0.0 | 9.5 | 6.9 | 2.1 | 0.0 | 0.0 | 0.0 | 7.4 |
| Fox Sparrow | *Passerella iliaca* | GEN | SDM | 4.3 | 2.3 | 0.0 | 0.0 | 3.4 | 6.2 | 4.3 | 17.4 | 0.0 | 3.7 |
| Three-toed Woodpecker | *Picoides dorsalis* | MAF | RES | 3.8 | 6.8 | 0.0 | 9.5 | 6.9 | 1.0 | 4.3 | 0.0 | 0.0 | 0.0 |
| Common Yellowthroat | *Geothlypis trichas* | SHR | NEO | 2.7 | 3.4 | 0.0 | 4.8 | 3.4 | 2.1 | 8.7 | 0.0 | 0.0 | 0.0 |
| Olive-sided Flycatcher | *Contopus cooperi* | SHR | NEO | 2.7 | 2.3 | 0.0 | 4.8 | 1.7 | 3.1 | 4.3 | 0.0 | 8.3 | 0.0 |
| Swamp Sparrow | *Melospiza georgiana* | SHR | SDM | 2.7 | 4.6 | 0.0 | 4.8 | 5.2 | 1.0 | 0.0 | 0.0 | 4.2 | 0.0 |
| Wilson's Warbler | *Wilsonia pusilla* | SHR | NEO | 2.7 | 2.3 | 0.0 | 0.0 | 3.4 | 3.1 | 4.3 | 4.3 | 4.2 | 0.0 |
| Black-capped Chickadee | *Poecile atricapillus* | GEN | RES | 2.2 | 0.0 | 0.0 | 0.0 | 0.0 | 4.1 | 0.0 | 4.3 | 4.2 | 7.4 |
| Ovenbird | *Seiurus aurocapilla* | MAF | NEO | 2.2 | 0.0 | 0.0 | 0.0 | 0.0 | 4.1 | 4.3 | 4.3 | 4.2 | 3.7 |
| Veery | *Catharus fuscescens* | YOF | NEO | 2.2 | 2.3 | 11.1 | 4.8 | 0.0 | 2.1 | 0.0 | 8.7 | 0.0 | 0.0 |
| American Crow | *Corvus brachyrhynchos* | GEN | SDM | 1.6 | 2.3 | 0.0 | 4.8 | 1.7 | 1.0 | 0.0 | 0.0 | 0.0 | 3.7 |
| Blue-headed Vireo | *Vireo solitarius* | MAF | NEO | 1.6 | 1.1 | 0.0 | 0.0 | 1.7 | 2.1 | 0.0 | 8.7 | 0.0 | 0.0 |
| Cedar Waxwing | *Bombycilla cedrorum* | YOF | SDM | 1.6 | 1.1 | 0.0 | 0.0 | 1.7 | 2.1 | 4.3 | 0.0 | 4.2 | 0.0 |
| Downy Woodpecker | *Picoides pubescens* | YOF | RES | 1.6 | 1.1 | 0.0 | 4.8 | 0.0 | 2.1 | 8.7 | 0.0 | 0.0 | 0.0 |
| Palm Warbler | *Dendroica palmarum* | SHR | NEO | 1.6 | 2.3 | 0.0 | 4.8 | 1.7 | 1.0 | 4.3 | 0.0 | 0.0 | 0.0 |
| Yellow-bellied Sapsucker | *Sphyrapicus varius* | MAF | SDM | 1.6 | 3.4 | 11.1 | 4.8 | 1.7 | 0.0 | 0.0 | 0.0 | 0.0 | 0.0 |
| Black-throated Blue Warbler | *Dendroica caerulescens* | MAF | NEO | 1.1 | 0.0 | 0.0 | 0.0 | 0.0 | 2.1 | 0.0 | 0.0 | 0.0 | 7.4 |
| Chestnut-sided Warbler | *Dendroica pensylvanica* | YOF | NEO | 1.1 | 2.3 | 0.0 | 0.0 | 3.4 | 0.0 | 0.0 | 0.0 | 0.0 | 0.0 |
| Grey-cheeked Thrush | *Catharus minimus* | MAF | NEO | 1.1 | 0.0 | 0.0 | 0.0 | 0.0 | 2.1 | 0.0 | 8.7 | 0.0 | 0.0 |
| Northern Flicker | *Colaptes auratus* | GEN | SDM | 1.1 | 2.3 | 0.0 | 0.0 | 3.4 | 0.0 | 0.0 | 0.0 | 0.0 | 0.0 |
| Philadelphia Vireo | *Vireo philadelphicus* | YOF | NEO | 1.1 | 2.3 | 0.0 | 4.8 | 1.7 | 0.0 | 0.0 | 0.0 | 0.0 | 0.0 |
| Spruce Grouse | *Falcipennis canadensis* | MAF | RES | 1.1 | 1.1 | 0.0 | 4.8 | 0.0 | 1.0 | 0.0 | 0.0 | 0.0 | 3.7 |
| Yellow Warbler | *Dendroica petechia* | SHR | NEO | 1.1 | 2.3 | 0.0 | 0.0 | 3.4 | 0.0 | 0.0 | 0.0 | 0.0 | 0.0 |
| American Kestrel | *Falco sparverius* | SHR | SDM | 0.5 | 1.1 | 0.0 | 0.0 | 1.7 | 0.0 | 0.0 | 0.0 | 0.0 | 0.0 |
| American Woodcock | *Scolopax minor* | MAF | SDM | 0.5 | 1.1 | 0.0 | 4.8 | 0.0 | 0.0 | 0.0 | 0.0 | 0.0 | 0.0 |
| Black-and-white Warbler | *Mniotilta varia* | YOF | NEO | 0.5 | 1.1 | 0.0 | 0.0 | 1.7 | 0.0 | 0.0 | 0.0 | 0.0 | 0.0 |
| Common Grackle | *Quiscalus quiscula* | GEN | SDM | 0.5 | 0.0 | 0.0 | 0.0 | 0.0 | 1.0 | 0.0 | 4.3 | 0.0 | 0.0 |
| Common Redpoll | *Carduelis flammea* | GEN | SDM | 0.5 | 0.0 | 0.0 | 0.0 | 0.0 | 1.0 | 0.0 | 0.0 | 0.0 | 3.7 |
| Ruffed Grouse | *Bonasa umbellus* | GEN | RES | 0.5 | 0.0 | 0.0 | 0.0 | 0.0 | 1.0 | 0.0 | 0.0 | 0.0 | 3.7 |
| Grey Catbird | *Dumetella carolinensis* | SHR | NEO | 0.5 | 1.1 | 0.0 | 0.0 | 1.7 | 0.0 | 0.0 | 0.0 | 0.0 | 0.0 |
| Hairy Woodpecker | *Picoides villosus* | MAF | RES | 0.5 | 1.1 | 0.0 | 0.0 | 1.7 | 0.0 | 0.0 | 0.0 | 0.0 | 0.0 |
| Mourning Dove | *Zenaida macroura* | GEN | SDM | 0.5 | 1.1 | 0.0 | 0.0 | 1.7 | 0.0 | 0.0 | 0.0 | 0.0 | 0.0 |
| Mourning Warbler | *Oporornis philadelphia* | YOF | NEO | 0.5 | 0.0 | 0.0 | 0.0 | 0.0 | 1.0 | 0.0 | 4.3 | 0.0 | 0.0 |
| Orange-crowned Warbler | *Vermivora celata* | YOF | NEO | 0.5 | 1.1 | 0.0 | 0.0 | 1.7 | 0.0 | 0.0 | 0.0 | 0.0 | 0.0 |
| Pileated Woodpecker | *Dryocopus pileatus* | MAF | RES | 0.5 | 0.0 | 0.0 | 0.0 | 0.0 | 1.0 | 0.0 | 0.0 | 4.2 | 0.0 |
| Purple Finch | *Carpodacus purpureus* | MAF | SDM | 0.5 | 1.1 | 11.1 | 0.0 | 0.0 | 0.0 | 0.0 | 0.0 | 0.0 | 0.0 |
| Whip-poor-will | *Caprimulgus vociferus* | YOF | NEO | 0.5 | 1.1 | 0.0 | 0.0 | 1.7 | 0.0 | 0.0 | 0.0 | 0.0 | 0.0 |

^a^ Habitat association: MAF: mature forest species, YOF: young forest species, SHR: shrub land species, and GEN: generalists.

^b^ Migratory status: RES: residents, SDM: short-distance migrants, and NEO: Neotropical migrants.

^c^ Age classes: N-I, N-II, and N-III stand for 50-79, 80-120, and >120 year old natural stands, respectively, C-1, C-2, C-3, and C-4 stand for 5-19, 20-39, 40-59, 60-70 year old clearcutting stands, respectively.
