# Supplementary material for: Landscape-Scale Disturbances Modified Bird Community Dynamics in Successional Forest Environment
Source: PLoS One. 2013 Nov 25;8(11):e81358. doi: 10.1371/journal.pone.0081358 (PMC3839899; doi:10.1371/journal.pone.0081358)
Supplement: Table S2 — The relative importance and independent contribution of environmental variables in explaining the variance of total- and group-richness calculated from (a) all data and (b) 60- to 70-year-old clearcutting stands and 50- to 79-year-old natural stands only. (DOCX) [file pone.0081358.s002.docx]

| (a) | **All species** | | | **Mature forest species** | | | **Young forest species** | | | **Shrub land species** | | |
| --- | --- | --- | --- | --- | --- | --- | --- | --- | --- | --- | --- | --- |
|  | *β* ^1^ | W^2^ | % var^3^ | *β* | W | % var | *β* | W | % var | *β* | W | % var |
| Latitude |  | 0.24 | 14.0 |  | 0.03 | 16.0 |  | 0.07 | 13.2 |  | 0.34 | 9.7 |
| Longitude |  | 0.05 |  |  | 0.31 |  |  | 0.13 |  |  | 0.94 |  |
| Basal area of conifer |  | 0.81 | 13.8 |  | 0.52 | 22.3 |  | 0.06 | 44.6 |  | 0.07 | 77.8 |
| Canopy cover |  | 0.38 |  |  | 0.74 |  | **-** | **1.00** |  | **-** | **1.00** |  |
| Sapling density |  | 0.06 |  |  | 0.07 |  |  | 0.37 |  |  | 0.05 |  |
| Basal area of snag |  | 0.68 |  |  | 0.66 |  | **-** | **1.00** |  |  | 0.94 |  |
| % disturb 200 m | **-** | **1.00** | 72.2 | **-** | **1.00** | 61.8 | **-** | **1.00** | 42.2 |  | 0.21 | 12.5 |
| % disturb 500 m |  | 0.06 |  |  | 0.08 |  |  | 0.31 |  |  | 0.23 |  |
| % disturb 1 km |  | 0.05 |  |  | 0.17 |  |  | 0.21 |  |  | 0.34 |  |
| % disturb 2 km |  | 0.05 |  |  | 0.17 |  |  | 0.06 |  |  | 0.06 |  |

| (a) cont’d | **Generalists** | | | **Residents** | | | **Short-distance migrants** | | | **Neotropical migrants** | | |
| --- | --- | --- | --- | --- | --- | --- | --- | --- | --- | --- | --- | --- |
|  | *β* | W | % var | *β* | W | % var | *β* | W | % var | *β* | W | % var |
| Latitude | **-** | **1.00** | 23.8 |  | 0.08 | 26.0 |  | 0.04 | 15.6 | **-** | **1.00** | 31.6 |
| Longitude |  | 0.15 |  | **-** | **1.00** |  |  | 0.44 |  |  | 0.22 |  |
| Basal area of conifer | **-** | **1.00** | 49.0 |  | 0.06 | 12.5 |  | 0.04 | 11.3 | **-** | **1.00** | 37.2 |
| Canopy cover |  | 0.05 |  |  | 0.06 |  | **-** | **1.00** |  |  | 0.04 |  |
| Sapling density |  | 0.05 |  |  | 0.06 |  |  | 0.25 |  |  | 0.08 |  |
| Basal area of snag |  | 0.94 |  |  | 0.79 |  |  | 0.42 |  |  | 0.04 |  |
| % disturb 200 m |  | 0.45 | 27.2 | **-** | **1.00** | 61.5 | **-** | **1.00** | 73.1 |  | 0.47 | 31.2 |
| % disturb 500 m |  | 0.34 |  |  | 0.40 |  |  | 0.08 |  |  | 0.26 |  |
| % disturb 1 km |  | 0.18 |  |  | 0.43 |  |  | 0.05 |  |  | 0.17 |  |
| % disturb 2 km |  | 0.14 |  |  | 0.21 |  |  | 0.32 |  |  | 0.40 |  |

| (b) | **All species** | | | **Mature forest species** | | | **Young forest species** | | | **Shrub land species** | | |
| --- | --- | --- | --- | --- | --- | --- | --- | --- | --- | --- | --- | --- |
|  | *β* | W | % var | *β* | W | % var | *β* | W | % var | *β* | W | % var |
| Latitude | + | **1.00** | 18.2 |  | 0.04 | 26.2 |  | 0.07 | 9.7 | **-** | **0.95** | 12.4 |
| Longitude |  | 0.15 |  | **-** | **1.00** |  |  | 0.03 |  |  | 0.31 |  |
| Basal area of conifer |  | 0.30 | 24.6 |  | 0.17 | 14.4 |  | 0.67 | 36.8 | **-** | **1.00** | 54.4 |
| Canopy cover | **-** | **1.00** |  |  | 0.30 |  |  | 0.55 |  |  | 0.19 |  |
| Sapling density |  | 0.26 |  |  | 0.04 |  |  | 0.55 |  | + | **0.95** |  |
| Basal area of snag |  | 0.11 |  |  | 0.04 |  |  | 0.06 |  | + | **1.00** |  |
| % disturb 200 m |  | 0.45 | 57.2 |  | 0.45 | 59.5 |  | 0.81 | 53.5 |  | 0.12 | 33.2 |
| % disturb 500 m | **-** | **1.00** |  |  | 0.29 |  | **-** | **1.00** |  |  | 0.84 |  |
| % disturb 1 km |  | 0.06 |  |  | 0.75 |  |  | 0.12 |  |  | 0.23 |  |
| % disturb 2 km |  | 0.06 |  |  | 0.25 |  |  | 0.13 |  |  | 0.59 |  |

| (b) cont’d | **Generalists** | | | **Residents** | | | **Short-distance migrants** | | | **Neotropical migrants** | | |
| --- | --- | --- | --- | --- | --- | --- | --- | --- | --- | --- | --- | --- |
|  | *β* | W | % var | *β* | W | % var | *β* | W | % var | *β* | W | % var |
| Latitude | **-** | **1.00** | 40.9 |  | 0.06 | 26.2 |  | 0.31 | 8.4 | **-** | **1.00** | 39.3 |
| Longitude |  | 0.15 |  | **-** | **1.00** |  |  | 0.08 |  |  | 0.06 |  |
| Basal area of conifer |  | 0.07 | 36.2 |  | 0.06 | 11.2 | **-** | **1.00** | 38.5 |  | 0.06 | 5.8 |
| Canopy cover | **-** | **1.00** |  |  | 0.07 |  | **-** | **1.00** |  |  | 0.17 |  |
| Sapling density |  | 0.67 |  |  | 0.07 |  |  | 0.12 |  |  | 0.06 |  |
| Basal area of snag |  | 0.21 |  |  | 0.19 |  |  | 0.23 |  |  | 0.14 |  |
| % disturb 200 m |  | 0.07 | 22.9 |  | 0.07 | 62.6 | **-** | **1.00** | 53.1 |  | 0.14 | 54.9 |
| % disturb 500 m | + | **1.00** |  |  | 0.17 |  | **-** | **1.00** |  | **-** | **1.00** |  |
| % disturb 1 km |  | 0.08 |  | **-** | **1.00** |  |  | 0.13 |  |  | 0.55 |  |
| % disturb 2 km |  | 0.08 |  |  | 0.29 |  |  | 0.30 |  |  | 0.65 |  |

1. *β* is the model averaged estimate of the corresponding parameter in the Poisson regress between species richness and environmental variables. Only the signs of significant estimates are shown here.
2. W is the model averaging importance. Variables with model averaging importance great than or equal to 0.95 are considered as important explanatory variables and are highlighted in bold.
3. % var is the relative independent contribution of each set of environmental variables in explaining the variance of species richness.
