# Supplementary material for: Landscape-Scale Disturbances Modified Bird Community Dynamics in Successional Forest Environment
Source: PLoS One. 2013 Nov 25;8(11):e81358. doi: 10.1371/journal.pone.0081358 (PMC3839899; doi:10.1371/journal.pone.0081358)
Supplement: Table S3 — The basic information of models used in model averaging and hierarchical partitioning procedures for (a) all data and (b) 60- to 70-year-old clearcutting stands and 50- to 79-year-old natural stands only. (DOCX) [file pone.0081358.s003.docx]

| (a) | | | | | |
| --- | --- | --- | --- | --- | --- |
| **Model**^*^ | **Degree of freedom** | **Log likelihood** | **AIC** | **ΔAIC** | **Model weight** |
| All species | | | | | |
| 127 | 5 | -20.98 | 51.95 | 0 | 0.031 |
| 17 | 4 | -22.49 | 52.97 | 1.02 | 0.019 |
| 1237 | 6 | -20.52 | 53.04 | 1.09 | 0.018 |
| 1247 | 6 | -20.59 | 53.17 | 1.22 | 0.017 |
| 37 | 4 | -22.60 | 53.21 | 1.26 | 0.017 |
| 347 | 5 | -21.78 | 53.55 | 1.60 | 0.014 |
| 12710 | 6 | -20.79 | 53.59 | 1.64 | 0.014 |
| 237 | 5 | -21.86 | 53.71 | 1.76 | 0.013 |
| 1279 | 6 | -20.89 | 53.79 | 1.84 | 0.012 |
| 1267 | 6 | -20.90 | 53.81 | 1.86 | 0.012 |
| 147 | 5 | -21.93 | 53.86 | 1.91 | 0.012 |
| 1278 | 6 | -20.96 | 53.92 | 1.97 | 0.012 |
| 12347 | 7 | -19.97 | 53.93 | 1.98 | 0.012 |
| 1257 | 6 | -20.97 | 53.94 | 1.99 | 0.012 |
| 137 | 5 | -21.97 | 53.94 | 1.99 | 0.012 |
| 2347 | 6 | -21.15 | 54.30 | 2.35 | 0.010 |
| 1347 | 6 | -21.21 | 54.42 | 2.47 | 0.009 |
| 1710 | 5 | -22.33 | 54.66 | 2.71 | 0.008 |
| 124710 | 7 | -20.38 | 54.75 | 2.80 | 0.008 |
| 12367 | 7 | -20.38 | 54.77 | 2.81 | 0.008 |
| Mature forest species | | | | | |
| 2371 | 5 | -68.60 | 147.20 | 0 | 0.019 |
| 2357 | 6 | -67.78 | 147.55 | 0.35 | 0.016 |
| 12371 | 6 | -67.92 | 147.84 | 0.64 | 0.013 |
| 1371 | 5 | -68.94 | 147.87 | 0.68 | 0.013 |
| 1357 | 6 | -67.94 | 147.89 | 0.69 | 0.013 |
| 171 | 4 | -69.96 | 147.92 | 0.72 | 0.013 |
| 1271 | 5 | -69.04 | 148.07 | 0.88 | 0.012 |
| 157 | 5 | -69.12 | 148.24 | 1.05 | 0.011 |
| 12357 | 7 | -67.13 | 148.26 | 1.07 | 0.011 |
| 236789 | 8 | -66.23 | 148.46 | 1.27 | 0.010 |
| 357 | 5 | -69.27 | 148.54 | 1.35 | 0.009 |
| 23710 | 6 | -68.35 | 148.70 | 1.50 | 0.009 |
| 12571 | 6 | -68.38 | 148.77 | 1.57 | 0.008 |
| 371 | 4 | -70.42 | 148.84 | 1.64 | 0.008 |
| 2378 | 6 | -68.49 | 148.98 | 1.78 | 0.008 |
| 2367 | 6 | -68.53 | 149.07 | 1.87 | 0.007 |
| 23678 | 7 | -67.55 | 149.09 | 1.90 | 0.007 |
| 1678 | 6 | -68.55 | 149.11 | 1.91 | 0.007 |
| 2379 | 6 | -68.56 | 149.12 | 1.92 | 0.007 |
| 23471 | 6 | -68.56 | 149.12 | 1.93 | 0.007 |
| Young forest species | | | | | |
| 2372 | 5 | -100.57 | 211.13 | 0 | 0.038 |
| 237101 | 6 | -99.68 | 211.37 | 0.23 | 0.034 |
| 23791 | 6 | -100.13 | 212.26 | 1.13 | 0.022 |
| 2367910 | 8 | -98.26 | 212.52 | 1.39 | 0.019 |
| 237910 | 7 | -99.30 | 212.60 | 1.47 | 0.018 |
| 23472 | 6 | -100.45 | 212.90 | 1.76 | 0.016 |
| 23679 | 7 | -99.46 | 212.91 | 1.78 | 0.016 |
| 235710 | 7 | -99.46 | 212.91 | 1.78 | 0.016 |
| 12372 | 6 | -100.47 | 212.94 | 1.81 | 0.016 |
| 23571 | 6 | -100.48 | 212.95 | 1.82 | 0.015 |
| 23781 | 6 | -100.49 | 212.99 | 1.85 | 0.015 |
| 23671 | 6 | -100.56 | 213.11 | 1.98 | 0.014 |
| 234710 | 7 | -99.57 | 213.14 | 2.01 | 0.014 |
| 123710 | 7 | -99.63 | 213.25 | 2.12 | 0.013 |
| 237810 | 7 | -99.68 | 213.36 | 2.22 | 0.013 |
| 236710 | 7 | -99.68 | 213.36 | 2.23 | 0.013 |
| 23479 | 7 | -99.90 | 213.80 | 2.67 | 0.010 |
| 2367891 | 8 | -98.91 | 213.83 | 2.70 | 0.010 |
| 23678910 | 9 | -97.92 | 213.84 | 2.70 | 0.010 |
| 12367910 | 9 | -97.94 | 213.89 | 2.75 | 0.010 |
| Shrub land species | | | | | |
| 235 | 5 | -105.74 | 221.49 | 0 | 0.020 |
| 23569 | 7 | -104.00 | 222.01 | 0.52 | 0.015 |
| 234569 | 8 | -103.11 | 222.22 | 0.73 | 0.014 |
| 2345 | 6 | -105.12 | 222.23 | 0.74 | 0.014 |
| 2356 | 6 | -105.28 | 222.55 | 1.06 | 0.012 |
| 1235 | 6 | -105.29 | 222.57 | 1.08 | 0.012 |
| 23457 | 7 | -104.30 | 222.60 | 1.11 | 0.011 |
| 2358 | 6 | -105.38 | 222.77 | 1.28 | 0.010 |
| 35 | 4 | -107.49 | 222.98 | 1.49 | 0.009 |
| 234 | 5 | -106.52 | 223.05 | 1.56 | 0.009 |
| 2359 | 6 | -105.66 | 223.32 | 1.83 | 0.008 |
| 23567 | 7 | -104.72 | 223.44 | 1.95 | 0.007 |
| 23572 | 6 | -105.72 | 223.44 | 1.95 | 0.007 |
| 23510 | 6 | -105.72 | 223.45 | 1.96 | 0.007 |
| 234567 | 8 | -103.74 | 223.49 | 2.00 | 0.007 |
| 3457 | 6 | -105.75 | 223.50 | 2.01 | 0.007 |
| 23459 | 7 | -104.77 | 223.55 | 2.06 | 0.007 |
| 123569 | 8 | -103.78 | 223.56 | 2.07 | 0.007 |
| 2356910 | 8 | -103.81 | 223.62 | 2.13 | 0.007 |
| 3 | 3 | -108.82 | 223.64 | 2.16 | 0.007 |
| Generalists | | | | | |
| 12471 | 6 | -73.84 | 159.68 | 0 | 0.030 |
| 124 | 5 | -74.87 | 159.75 | 0.07 | 0.029 |
| 1249 | 6 | -73.94 | 159.88 | 0.20 | 0.027 |
| 12469 | 7 | -73.21 | 160.42 | 0.74 | 0.021 |
| 12457 | 7 | -73.65 | 161.30 | 1.62 | 0.013 |
| 12489 | 7 | -73.68 | 161.36 | 1.68 | 0.013 |
| 123471 | 7 | -73.70 | 161.39 | 1.71 | 0.013 |
| 1246 | 6 | -74.71 | 161.42 | 1.74 | 0.013 |
| 12479 | 7 | -73.75 | 161.51 | 1.83 | 0.012 |
| 1247101 | 7 | -73.76 | 161.53 | 1.85 | 0.012 |
| 12459 | 7 | -73.77 | 161.55 | 1.87 | 0.012 |
| 1248 | 6 | -74.78 | 161.55 | 1.87 | 0.012 |
| 12478 | 7 | -73.80 | 161.59 | 1.91 | 0.012 |
| 1245 | 6 | -74.81 | 161.62 | 1.94 | 0.011 |
| 12467 | 7 | -73.81 | 161.62 | 1.94 | 0.011 |
| 1471 | 5 | -75.83 | 161.66 | 1.98 | 0.011 |
| 12349 | 7 | -73.84 | 161.68 | 2.00 | 0.011 |
| 1234 | 6 | -74.85 | 161.70 | 2.02 | 0.011 |
| 12410 | 6 | -74.85 | 161.71 | 2.03 | 0.011 |
| 124910 | 7 | -73.86 | 161.73 | 2.05 | 0.011 |
| Residents | | | | | |
| 257 | 5 | -117.89 | 245.79 | 0 | 0.040 |
| 25679 | 7 | -116.14 | 246.27 | 0.48 | 0.031 |
| 5679 | 6 | -117.47 | 246.93 | 1.15 | 0.022 |
| 2567 | 6 | -117.48 | 246.97 | 1.18 | 0.022 |
| 256789 | 8 | -115.51 | 247.02 | 1.23 | 0.021 |
| 2457 | 6 | -117.58 | 247.15 | 1.36 | 0.020 |
| 56789 | 7 | -116.74 | 247.47 | 1.68 | 0.017 |
| 2578 | 6 | -117.78 | 247.56 | 1.77 | 0.016 |
| 57 | 4 | -119.79 | 247.59 | 1.80 | 0.016 |
| 12572 | 6 | -117.84 | 247.69 | 1.90 | 0.015 |
| 23573 | 6 | -117.87 | 247.75 | 1.96 | 0.015 |
| 2579 | 6 | -117.89 | 247.77 | 1.99 | 0.015 |
| 25710 | 6 | -117.89 | 247.78 | 2.00 | 0.015 |
| 245679 | 8 | -115.98 | 247.97 | 2.18 | 0.013 |
| 2567910 | 8 | -116.12 | 248.24 | 2.45 | 0.012 |
| 125679 | 8 | -116.13 | 248.27 | 2.48 | 0.011 |
| 235679 | 8 | -116.14 | 248.27 | 2.48 | 0.011 |
| 1571 | 5 | -119.22 | 248.44 | 2.65 | 0.011 |
| 15679 | 7 | -117.24 | 248.47 | 2.68 | 0.010 |
| 24567 | 7 | -117.31 | 248.62 | 2.83 | 0.010 |
| Short-distance migrants | | | | | |
| 372 | 4 | -62.34 | 132.68 | 0 | 0.024 |
| 3571 | 5 | -61.67 | 133.35 | 0.67 | 0.017 |
| 2373 | 5 | -61.73 | 133.45 | 0.78 | 0.016 |
| 378 | 5 | -61.77 | 133.53 | 0.86 | 0.016 |
| 3710 | 5 | -61.86 | 133.71 | 1.04 | 0.014 |
| 3578 | 6 | -60.86 | 133.72 | 1.04 | 0.014 |
| 23574 | 6 | -60.86 | 133.72 | 1.04 | 0.014 |
| 23578 | 7 | -59.87 | 133.75 | 1.07 | 0.014 |
| 35710 | 6 | -60.96 | 133.92 | 1.25 | 0.013 |
| 2357101 | 7 | -59.99 | 133.99 | 1.31 | 0.012 |
| 23782 | 6 | -61.07 | 134.14 | 1.46 | 0.011 |
| 367 | 5 | -62.13 | 134.26 | 1.58 | 0.011 |
| 237102 | 6 | -61.17 | 134.34 | 1.67 | 0.010 |
| 235789 | 8 | -59.23 | 134.47 | 1.79 | 0.010 |
| 3471 | 5 | -62.26 | 134.51 | 1.84 | 0.010 |
| 1372 | 5 | -62.30 | 134.60 | 1.93 | 0.009 |
| 2357810 | 8 | -59.30 | 134.61 | 1.93 | 0.009 |
| 379 | 5 | -62.34 | 134.67 | 2.00 | 0.009 |
| 357810 | 7 | -60.40 | 134.79 | 2.12 | 0.008 |
| 35789 | 7 | -60.41 | 134.83 | 2.15 | 0.008 |
| Neotropical migrants | | | | | |
| 1472 | 5 | -67.42 | 144.83 | 0 | 0.027 |
| 148 | 5 | -67.49 | 144.98 | 0.15 | 0.025 |
| 149 | 5 | -67.66 | 145.33 | 0.50 | 0.021 |
| 1457 | 6 | -66.67 | 145.35 | 0.51 | 0.021 |
| 1478 | 6 | -66.94 | 145.89 | 1.05 | 0.016 |
| 14 | 4 | -68.97 | 145.93 | 1.10 | 0.016 |
| 1459 | 6 | -67.00 | 146.00 | 1.16 | 0.015 |
| 14689 | 7 | -66.16 | 146.32 | 1.48 | 0.013 |
| 1458 | 6 | -67.17 | 146.34 | 1.51 | 0.013 |
| 14710 | 6 | -67.19 | 146.37 | 1.54 | 0.012 |
| 146 | 5 | -68.24 | 146.49 | 1.65 | 0.012 |
| 1468 | 6 | -67.29 | 146.59 | 1.75 | 0.011 |
| 1489 | 6 | -67.33 | 146.66 | 1.82 | 0.011 |
| 1479 | 6 | -67.34 | 146.67 | 1.84 | 0.011 |
| 14810 | 6 | -67.35 | 146.69 | 1.86 | 0.011 |
| 14578 | 7 | -66.35 | 146.71 | 1.88 | 0.011 |
| 1467 | 6 | -67.38 | 146.75 | 1.92 | 0.010 |
| 13471 | 6 | -67.38 | 146.77 | 1.94 | 0.010 |
| 12472 | 6 | -67.42 | 146.83 | 2.00 | 0.010 |
| 1348 | 6 | -67.42 | 146.85 | 2.01 | 0.010 |

| (b) | | | | | |
| --- | --- | --- | --- | --- | --- |
| **Model**^*^ | **Degree of freedom** | **Log likelihood** | **AIC** | **ΔAIC** | **Model weight** |
| All species | | | | | |
| 349 | 5 | -0.28 | 10.56 | 0 | 0.028 |
| 3479 | 6 | 0.52 | 10.95 | 0.39 | 0.023 |
| 34910 | 6 | 0.38 | 11.24 | 0.69 | 0.020 |
| 13479 | 7 | 1.20 | 11.61 | 1.05 | 0.016 |
| 1349 | 6 | 0.10 | 11.80 | 1.25 | 0.015 |
| 347910 | 7 | 1.04 | 11.93 | 1.37 | 0.014 |
| 3489 | 6 | -0.05 | 12.09 | 1.53 | 0.013 |
| 134910 | 7 | 0.93 | 12.14 | 1.58 | 0.013 |
| 3469 | 6 | -0.08 | 12.16 | 1.60 | 0.012 |
| 34579 | 7 | 0.91 | 12.18 | 1.62 | 0.012 |
| 1347910 | 8 | 1.88 | 12.23 | 1.67 | 0.012 |
| 2349 | 6 | -0.12 | 12.24 | 1.69 | 0.012 |
| 23479 | 7 | 0.85 | 12.30 | 1.74 | 0.012 |
| 3459 | 6 | -0.21 | 12.43 | 1.87 | 0.011 |
| 134579 | 8 | 1.73 | 12.55 | 1.99 | 0.010 |
| 234910 | 7 | 0.69 | 12.62 | 2.06 | 0.010 |
| 34789 | 7 | 0.63 | 12.73 | 2.18 | 0.009 |
| 13469 | 7 | 0.59 | 12.82 | 2.26 | 0.009 |
| 34679 | 7 | 0.56 | 12.89 | 2.33 | 0.009 |
| 2347910 | 8 | 1.53 | 12.93 | 2.38 | 0.008 |
| Mature forest species | | | | | |
| 56 | 4 | -4.24 | 16.49 | 0 | 0.019 |
| 567 | 5 | -3.54 | 17.07 | 0.58 | 0.014 |
| 58 | 4 | -4.59 | 17.18 | 0.69 | 0.014 |
| 1567 | 6 | -2.65 | 17.29 | 0.80 | 0.013 |
| 356 | 5 | -3.72 | 17.45 | 0.96 | 0.012 |
| 3567 | 6 | -2.73 | 17.46 | 0.97 | 0.012 |
| 156 | 5 | -3.79 | 17.58 | 1.09 | 0.011 |
| 358 | 5 | -3.89 | 17.78 | 1.30 | 0.010 |
| 5789 | 6 | -3.07 | 18.14 | 1.66 | 0.008 |
| 5679 | 6 | -3.08 | 18.17 | 1.68 | 0.008 |
| 569 | 5 | -4.09 | 18.18 | 1.70 | 0.008 |
| 15679 | 7 | -2.11 | 18.22 | 1.73 | 0.008 |
| 256 | 5 | -4.12 | 18.24 | 1.76 | 0.008 |
| 456 | 5 | -4.13 | 18.26 | 1.77 | 0.008 |
| 3579 | 6 | -3.13 | 18.26 | 1.77 | 0.008 |
| 568 | 5 | -4.14 | 18.29 | 1.80 | 0.008 |
| 5610 | 5 | -4.16 | 18.32 | 1.83 | 0.008 |
| 35789 | 7 | -2.17 | 18.33 | 1.85 | 0.008 |
| 35679 | 7 | -2.18 | 18.36 | 1.88 | 0.008 |
| 158 | 5 | -4.28 | 18.56 | 2.07 | 0.007 |
| Young forest species | | | | | |
| 17910 | 6 | -14.92 | 41.83 | 0 | 0.015 |
| 1379 | 6 | -15.20 | 42.41 | 0.58 | 0.011 |
| 137910 | 7 | -14.28 | 42.56 | 0.72 | 0.010 |
| 127910 | 7 | -14.34 | 42.68 | 0.84 | 0.010 |
| 37910 | 6 | -15.49 | 42.99 | 1.16 | 0.008 |
| 179 | 5 | -16.52 | 43.04 | 1.21 | 0.008 |
| 13789 | 7 | -14.55 | 43.10 | 1.27 | 0.008 |
| 379 | 5 | -16.63 | 43.26 | 1.42 | 0.007 |
| 7910 | 5 | -16.66 | 43.33 | 1.49 | 0.007 |
| 3679 | 6 | -15.67 | 43.35 | 1.51 | 0.007 |
| 3789 | 6 | -15.67 | 43.35 | 1.51 | 0.007 |
| 39 | 4 | -17.72 | 43.44 | 1.60 | 0.007 |
| 134791 | 7 | -14.74 | 43.48 | 1.64 | 0.006 |
| 1910 | 5 | -16.74 | 43.48 | 1.64 | 0.006 |
| 13679 | 7 | -14.74 | 43.49 | 1.65 | 0.006 |
| 910 | 4 | -17.83 | 43.66 | 1.83 | 0.006 |
| 178910 | 7 | -14.85 | 43.69 | 1.86 | 0.006 |
| 139 | 5 | -16.85 | 43.70 | 1.86 | 0.006 |
| 3910 | 5 | -16.88 | 43.76 | 1.92 | 0.006 |
| 167910 | 7 | -14.89 | 43.78 | 1.94 | 0.006 |
| Shrub land species | | | | | |
| 1248910 | 8 | -1.89 | 19.79 | 0 | 0.021 |
| 12458910 | 9 | -1.10 | 20.19 | 0.40 | 0.017 |
| 1245910 | 8 | -2.25 | 20.49 | 0.71 | 0.015 |
| 12348910 | 9 | -1.28 | 20.56 | 0.77 | 0.014 |
| 124910 | 7 | -3.32 | 20.63 | 0.85 | 0.014 |
| 12478910 | 9 | -1.53 | 21.05 | 1.27 | 0.011 |
| 1246910 | 8 | -2.62 | 21.24 | 1.45 | 0.010 |
| 124610 | 7 | -3.68 | 21.35 | 1.57 | 0.010 |
| 12456910 | 9 | -1.68 | 21.36 | 1.57 | 0.010 |
| 1.23E+08 | 10 | -0.74 | 21.49 | 1.70 | 0.009 |
| 1247910 | 8 | -2.80 | 21.60 | 1.81 | 0.009 |
| 124810 | 7 | -3.85 | 21.71 | 1.92 | 0.008 |
| 12468910 | 9 | -1.86 | 21.73 | 1.94 | 0.008 |
| 1238 | 6 | -4.89 | 21.77 | 1.99 | 0.008 |
| 1.23E+08 | 10 | -0.96 | 21.91 | 2.13 | 0.007 |
| 1.25E+08 | 10 | -0.98 | 21.95 | 2.17 | 0.007 |
| 123489 | 8 | -2.98 | 21.96 | 2.18 | 0.007 |
| 1234610 | 8 | -3.02 | 22.03 | 2.25 | 0.007 |
| 12346910 | 9 | -2.06 | 22.12 | 2.33 | 0.007 |
| 1.25E+08 | 10 | -1.07 | 22.15 | 2.36 | 0.007 |
| Generalists | | | | | |
| 349101 | 6 | -13.97 | 39.94 | 0 | 0.039 |
| 3491 | 5 | -15.05 | 40.09 | 0.15 | 0.036 |
| 2349101 | 7 | -13.32 | 40.65 | 0.71 | 0.028 |
| 23491 | 6 | -14.71 | 41.42 | 1.48 | 0.019 |
| 346910 | 7 | -13.74 | 41.48 | 1.54 | 0.018 |
| 348910 | 7 | -13.82 | 41.65 | 1.71 | 0.017 |
| 345910 | 7 | -13.87 | 41.74 | 1.80 | 0.016 |
| 34591 | 6 | -14.89 | 41.78 | 1.84 | 0.016 |
| 3479101 | 7 | -13.96 | 41.93 | 1.99 | 0.015 |
| 1349101 | 7 | -13.97 | 41.93 | 2.00 | 0.014 |
| 2346910 | 8 | -12.99 | 41.98 | 2.04 | 0.014 |
| 34691 | 6 | -15.00 | 42.00 | 2.06 | 0.014 |
| 2345910 | 8 | -13.01 | 42.02 | 2.08 | 0.014 |
| 13491 | 6 | -15.04 | 42.08 | 2.14 | 0.013 |
| 34791 | 6 | -15.04 | 42.08 | 2.15 | 0.013 |
| 34891 | 6 | -15.04 | 42.08 | 2.15 | 0.013 |
| 34710 | 6 | -15.12 | 42.24 | 2.30 | 0.012 |
| 2348910 | 8 | -13.19 | 42.39 | 2.45 | 0.012 |
| 347 | 5 | -16.21 | 42.42 | 2.48 | 0.011 |
| 23479101 | 8 | -13.32 | 42.64 | 2.71 | 0.010 |
| Residents | | | | | |
| 561 | 4 | -17.32 | 42.64 | 0 | 0.030 |
| 5681 | 5 | -16.66 | 43.31 | 0.67 | 0.022 |
| 2561 | 5 | -16.71 | 43.43 | 0.78 | 0.021 |
| 5689 | 6 | -15.96 | 43.92 | 1.27 | 0.016 |
| 5691 | 5 | -17.05 | 44.10 | 1.46 | 0.015 |
| 56101 | 5 | -17.16 | 44.31 | 1.67 | 0.013 |
| 2568 | 6 | -16.18 | 44.37 | 1.72 | 0.013 |
| 5671 | 5 | -17.20 | 44.39 | 1.75 | 0.013 |
| 3561 | 5 | -17.25 | 44.51 | 1.86 | 0.012 |
| 4561 | 5 | -17.29 | 44.59 | 1.94 | 0.012 |
| 1561 | 5 | -17.30 | 44.60 | 1.95 | 0.011 |
| 56810 | 6 | -16.37 | 44.74 | 2.10 | 0.011 |
| 5678 | 6 | -16.38 | 44.75 | 2.11 | 0.011 |
| 2569 | 6 | -16.52 | 45.04 | 2.40 | 0.009 |
| 4568 | 6 | -16.53 | 45.06 | 2.42 | 0.009 |
| 25610 | 6 | -16.56 | 45.11 | 2.47 | 0.009 |
| 3568 | 6 | -16.60 | 45.19 | 2.55 | 0.009 |
| 1568 | 6 | -16.62 | 45.24 | 2.60 | 0.008 |
| 2567 | 6 | -16.63 | 45.25 | 2.61 | 0.008 |
| 25689 | 7 | -15.64 | 45.28 | 2.64 | 0.008 |
| Short-distance migrants | | | | | |
| 13791 | 6 | -13.81 | 39.62 | 0 | 0.037 |
| 134792 | 7 | -13.07 | 40.15 | 0.53 | 0.028 |
| 137891 | 7 | -13.16 | 40.33 | 0.70 | 0.026 |
| 12379 | 7 | -13.25 | 40.49 | 0.87 | 0.024 |
| 13579 | 7 | -13.46 | 40.92 | 1.29 | 0.019 |
| 136789 | 8 | -12.63 | 41.25 | 1.63 | 0.016 |
| 123789 | 8 | -12.68 | 41.37 | 1.74 | 0.015 |
| 134789 | 8 | -12.69 | 41.38 | 1.76 | 0.015 |
| 13479101 | 8 | -12.71 | 41.41 | 1.79 | 0.015 |
| 123479 | 8 | -12.71 | 41.41 | 1.79 | 0.015 |
| 1379101 | 7 | -13.74 | 41.47 | 1.85 | 0.015 |
| 136791 | 7 | -13.77 | 41.54 | 1.92 | 0.014 |
| 1378910 | 8 | -12.84 | 41.68 | 2.06 | 0.013 |
| 1346789 | 9 | -11.93 | 41.86 | 2.24 | 0.012 |
| 135789 | 8 | -13.01 | 42.02 | 2.40 | 0.011 |
| 1345791 | 8 | -13.04 | 42.08 | 2.46 | 0.011 |
| 13478910 | 9 | -12.04 | 42.09 | 2.46 | 0.011 |
| 1237910 | 8 | -13.05 | 42.10 | 2.48 | 0.011 |
| 134679 | 8 | -13.07 | 42.14 | 2.52 | 0.010 |
| 123579 | 8 | -13.10 | 42.19 | 2.57 | 0.010 |
| Neotropical migrants | | | | | |
| 4689 | 6 | -5.45 | 22.91 | 0 | 0.029 |
| 49 | 4 | -7.57 | 23.15 | 0.24 | 0.026 |
| 489 | 5 | -6.88 | 23.77 | 0.86 | 0.019 |
| 34689 | 7 | -5.01 | 24.02 | 1.11 | 0.017 |
| 46789 | 7 | -5.06 | 24.12 | 1.21 | 0.016 |
| 3492 | 5 | -7.10 | 24.21 | 1.30 | 0.015 |
| 249 | 5 | -7.14 | 24.29 | 1.38 | 0.015 |
| 24689 | 7 | -5.31 | 24.63 | 1.72 | 0.012 |
| 479 | 5 | -7.41 | 24.83 | 1.92 | 0.011 |
| 14689 | 7 | -5.43 | 24.86 | 1.95 | 0.011 |
| 468910 | 7 | -5.43 | 24.86 | 1.96 | 0.011 |
| 45689 | 7 | -5.45 | 24.91 | 2.00 | 0.011 |
| 4910 | 5 | -7.47 | 24.95 | 2.04 | 0.010 |
| 34892 | 6 | -6.48 | 24.96 | 2.05 | 0.010 |
| 346789 | 8 | -4.49 | 24.98 | 2.07 | 0.010 |
| 459 | 5 | -7.53 | 25.05 | 2.14 | 0.010 |
| 2489 | 6 | -6.54 | 25.08 | 2.17 | 0.010 |
| 469 | 5 | -7.57 | 25.14 | 2.23 | 0.009 |
| 149 | 5 | -7.57 | 25.15 | 2.24 | 0.009 |
| 349102 | 6 | -6.58 | 25.16 | 2.25 | 0.009 |

*Model term code: 1: basal area of conifer, 2: basal area of snag, 3: canopy cover, 4: latitude, 5: longitude, 6: proportion of disturbed area at 1km, 7: proportion of disturbed area at 200m, 8: proportion of disturbed area at 2km, 9: proportion of disturbed area at 500m, and 10: sapling density.
